# Inhibition of anaerobic digestion by phenol and ammonia: Effect on degradation performances and microbial dynamics

**DOI:** 10.1016/j.dib.2018.06.119

**Published:** 2018-07-06

**Authors:** Simon Poirier, Olivier Chapleur

**Affiliations:** Hydrosystems and Bioprocesses Research Unit, Irstea, 1 rue Pierre-Gilles de Gennes, CS 10030, 92761 Antony Cedex, France

## Abstract

Data in this article provide detailed information on the microbial dynamics during inhibition of anaerobic digestion by phenol and ammonia. Ten concentrations of both inhibitors were tested in triplicates. Data include the operational conditions and degradation performance measurements, as well as microbial community analysis, by 16S rRNA gene sequencing, at different time points for the different conditions (96 samples). Sequencing data were generated by using IonTorrent PGM sequencer. This data is associated with the research articles “Community shifts within anaerobic digestion microbiota facing phenol inhibition: Towards early warning microbial indicators?” (Poirier et al., 2016a) [1] and “Anaerobic digestion of biowaste under extreme ammonia concentration: Identification of key microbial phylotypes” (Poirier et al., 2016b) [2]. The sequencing data have been deposited in the bioproject PRJNA450311, with the dataset identifier (TaxID) 1263854. Samples accession numbers go from SAMN08934853 to SAMN08934947.

**Specifications Table**

**Table:** 

Subject area	*Biology*
More specific subject area	*Microbial ecology of anaerobic digestion*
Type of data	*Table, figure, raw sequencing data*
How data was acquired	Gas production and composition were measured respectively by using a differential manometer (Digitron 2082P, Margam, UK) and a micro gas chromatography (CP4900, Varian, Palo Alto, USA). Volatile fatty acids concentrations were quantified by ionic chromatography coupled with conductometric detection (Dionex 120, ThermoFisher). DNA sequencing was carried out with Ion Torrent Personal Genome Machine.
Data format	Raw, analyzed
Experimental factors	Liquid samples were centrifuged (10,000×*g*, 10 min). Pellets and supernatants were stored separately at − 20 °C before respectively DNA extraction with Powersoil™DNA isolation kit (Mobio Laboratories Inc. Carlsbad) and dilution for VFA analysis.
Experimental features	60 anaerobic batch digesters were carried out to evaluate in triplicate the influence of ten different concentrations of ammonia and phenol on anaerobic digestion performances and microbial dynamics.
Data source location	*Antony, France*
Data accessibility	*Data are available in the article. The sequencing data have been deposited in the bioproject PRJNA450311, with the dataset identifier (TaxID) 1263854*

**Value of the data**

• This data provides a link between anaerobic digester performance (biogas production and volatile fatty acids accumulation), inhibition levels and microbial community composition.
• Sequencing data can be used to understand the variation of microbial community composition, abundance and diversity in anaerobic digesters inhibited by phenol or ammonia.
• Sequencing data can be used to identify micro-organisms characteristics of the different types and level of inhibition. Accessibility to 16S rRNA sequence data and detailed associated metadata allows researchers to perform new analyses with their own research purposes.
• A wide number of conditions were tested (20) in triplicates and in similar experimental system, with the same inoculum and feeding, at the same time. A very important number of samples were sequenced, at different time points.

## 1 Data

**Fig. 1:**
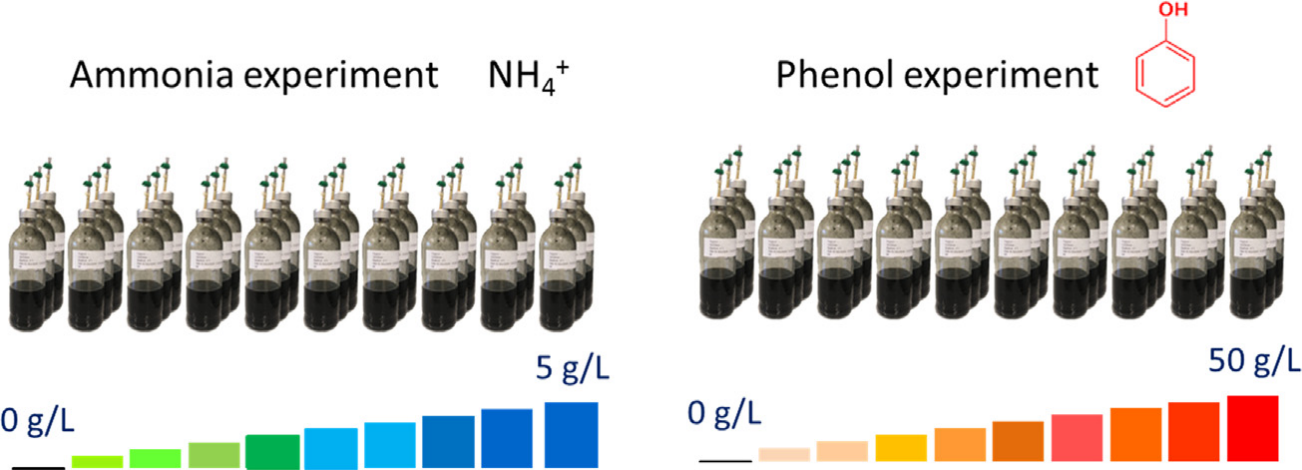
Experimental design.

**Fig. 2:**
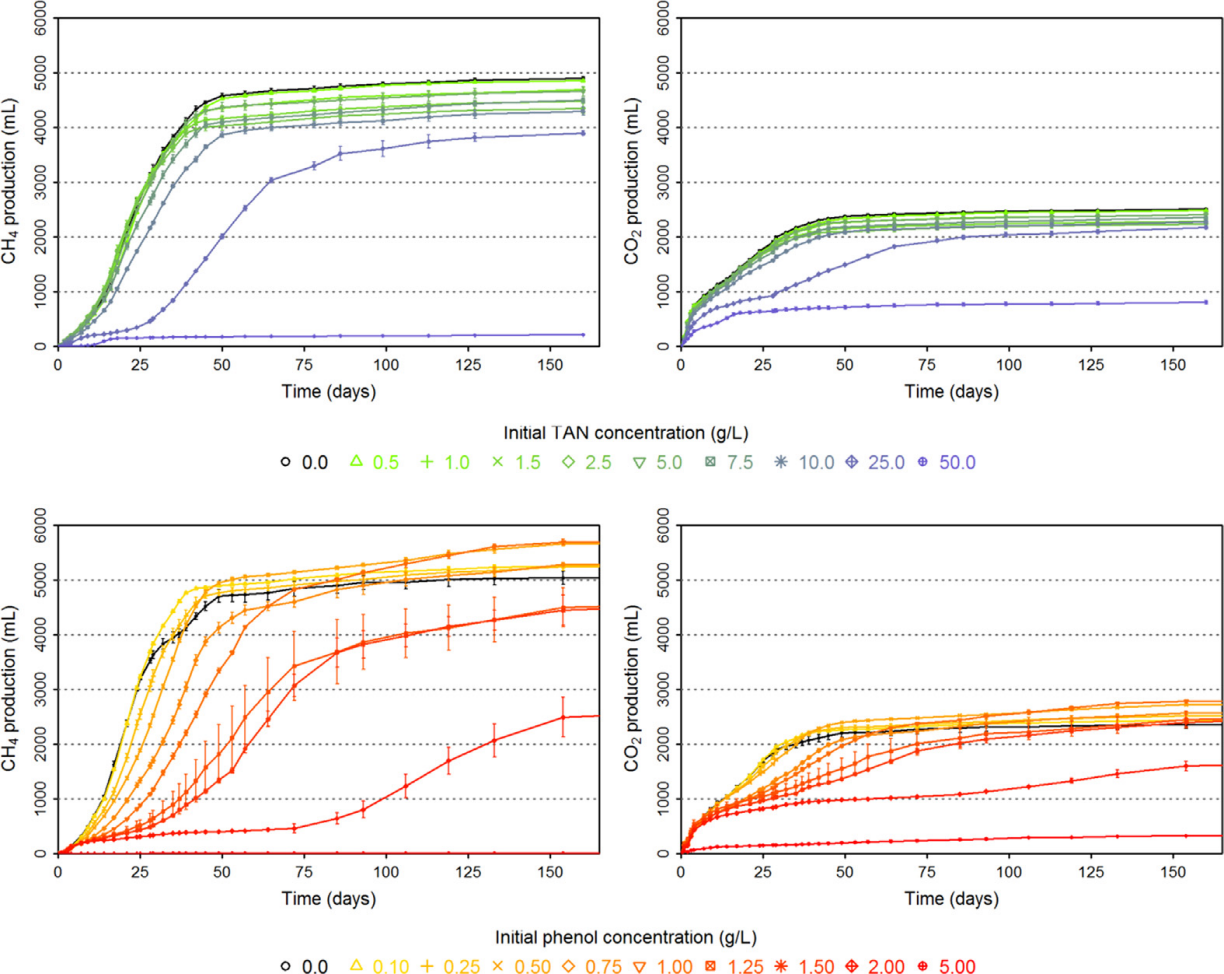
Cumulated CH_4_ and CO_2_ production (mL) over time (number of days) for the different level of ammonia and phenol initially added.

**Fig. 3:**
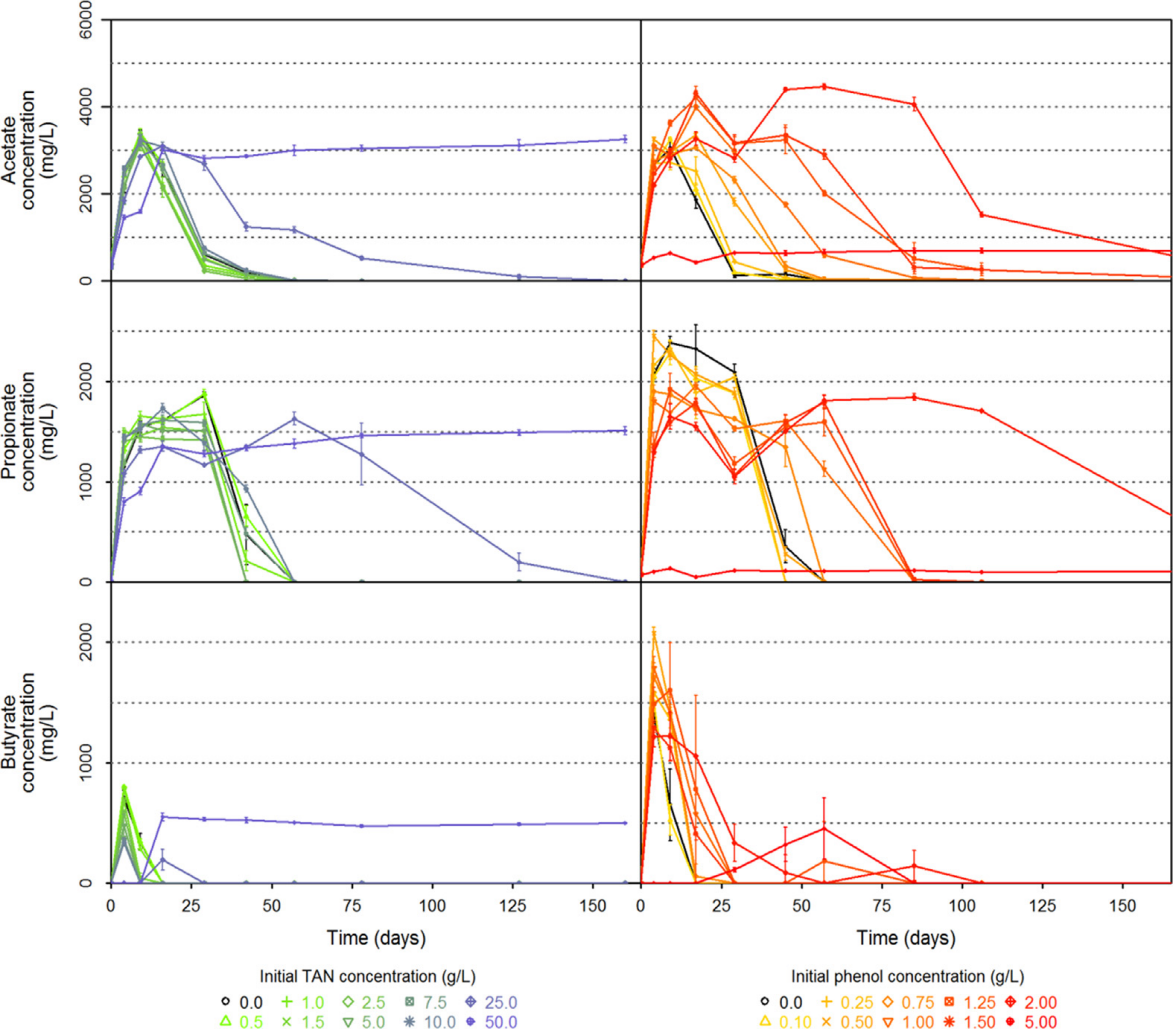
Volatile fatty acids concentrations (mg/L) over time (number of days) for the different level of ammonia and phenol initially added.

Fig. 1 illustrates the global experimental design of this study. Data presented in Supplementary Table 1 include inhibitor concentration for each digester, as well as the samples which were selected to sequence the V4–V5 region of 16S rRNA. Supplementary Tables 2–5 detail cumulated CH_4_ and CO_2_ production over time of each digester while Fig. 2 illustrates all these datasets (mean per triplicate of digesters). Similarly, Supplementary Tables 6–11 present the volatile fatty acids (acetate, propionate and butyrate) accumulation over time of each digester while Fig. 3 illustrates the same datasets (mean per triplicate of digesters).

Mean values of each triplicate of bioreactors are presented for CH_4_ and CO_2_ production and error bars represent standard deviation within triplicates. The black line represents the mean values in control bioreactors (no inhibitor) for CH_4_ and CO_2_ production.

Acetate, propionate and butyrate concentrations in the liquid phase over time, for the different groups of triplicate bioreactors (mean values, error bars represent standard deviation within triplicates). The black line represents the mean values in control bioreactors (no inhibitor) for volatile fatty acids concentration.

## 2 Experimental design, materials and methods

### 2.1 Experimental design and sampling

60 anaerobic batch bioreactors were initially seeded with 20 g of centrifuged methanogenic sludge as inoculum and supplemented with 50 g of mashed biowaste as substrate corresponding to an initial organic loading of 10 g COD/g COD. In a first set of 30 bioreactors, NH_4_Cl (99.998%, Sigma Aldrich) was added in order to reach 10 different ammonia concentrations (from 0.0 g/L up to 50.0 g/L). In the second set of 30 bioreactors, phenol (99%, ACROS Organics) was added in order to reach 10 different concentrations (from 0.0 g/L up to 5.0 g/L). Time zero (T0) samples were taken and all reactors were incubated without agitation, in the dark, at 35 °C. Liquid samples (2 mL) were periodically taken through the septum and centrifuged at 10,000×*g* for 10 min. Pellets were separated from the supernatant and stored at − 20 °C.

### 2.2 DNA extraction, amplification and sequencing

Total DNA was extracted from the pellet using PowersoilTM DNA isolation kit (Mobio Laboratories Inc. Carlsbad) according to the manufacturer׳s instructions. DNA extracts were used for the amplification of the bacterial and archaeal hypervariable region V4–V5 of the 16S rRNA genes with the primers 515F (5′-GTGYCAGCMGCCGCGGTA-3′) and 928R (5′-CCCCGYCAATTCMTTTRAGT-3′) as described in [1], [2]. Sequencing was performed on Ion Torrent Personal Genome Machine using Ion 316 chip and the Ion PGM Sequencing 400 Kit.

### 2.3 Sequence read processing

PGM software filtered out low quality and polyclonal sequence reads, and quality filtered data was exported as FastQ file.

## Appendix A Supplementary material

Supplementary material Table 1 Sample nomenclature. Table 2 Cumulated CH_4_ production (mL) over time (days) for the different levels of ammonia initially added. Table 3 Cumulated CH_4_ production (mL) over time for the different levels of phenol initially added. Table 4 Cumulated CO_2_ production (mL) over time for the different levels of ammonia initially added. Table 5 Cumulated CO_2_ production (mL) over time for the different levels of phenol initially added. Table 6 Acetate concentration (mg/L) over time (days) for the different levels of ammonia. Table 7 Acetate concentration (mg/L) over time (days) for the different levels of phenol. Table 8 Propionate concentrations (mg/L) over time (days) for the different levels of ammonia. Table 9 Propionate concentrations (mg/L) over time (days) for the different levels of phenol. Table 10 Butyrate concentrations (mg/L) over time (days) for the different levels of ammonia. Table 11 Butyrate concentrations (mg/L) over time (days) for the different levels of phenol.

## Footnotes

Appendix A Supplementary data associated with this article can be found in the online version at https://doi.org/10.1016/j.dib.2018.06.119.
